# Elongation factor-2 kinase is a critical determinant of the fate and antitumor immunity of CD8^+^ T cells

**DOI:** 10.1126/sciadv.abl9783

**Published:** 2022-02-02

**Authors:** Jugal Kishore Das, Yijie Ren, Anil Kumar, Hao-Yun Peng, Liqing Wang, Xiaofang Xiong, Robert C. Alaniz, Paul de Figueiredo, Xingcong Ren, Xiaoqi Liu, Alexey G. Ryazonov, Jin-Ming Yang, Jianxun Song

**Affiliations:** 1 Department of Microbial Pathogenesis and Immunology, Texas A&M University Health Science Center, Bryan, TX 77843, USA.; 2 Norman Borlaug Center, Texas A&M University, College Station, TX 77843, USA.; 3 Department of Veterinary Pathobiology, College of Veterinary Medicine, Texas A&M University, College Station, Texas 77843, USA.; 4 Department of Toxicology and Cancer Biology, Department of Pharmacology and Nutritional Science, and Markey Cancer Center, University of Kentucky College of Medicine, Lexington, KY 40536, USA.; 5 Department of Pharmacology, Rutgers Robert Wood Johnson Medical School, Piscataway, NJ 08854, USA.

## Abstract

eEF-2K has important roles in stress responses and cellular metabolism. We report here a previously unappreciated but critical role of eEF-2K in regulating the fate and cytocidal activity of CD8^+^ T cells. CD8^+^ T cells from eEF-2K KO mice were more proliferative but had lower survival than their wild-type counterparts after their activation, followed by occurrence of premature senescence and exhaustion. eEF-2K KO CD8^+^ T cells were more metabolically active and showed hyperactivation of the Akt-mTOR-S6K pathway. Loss of eEF-2K substantially impaired the activity of CD8^+^ T cells. Furthermore, the antitumor efficacy and tumor infiltration of the CAR-CD8^+^ T cells lacking eEF-2K were notably reduced as compared to the control CAR-CD8^+^ T cells. Thus, eEF-2K is critically required for sustaining the viability and function of cytotoxic CD8^+^ T cells, and therapeutic augmentation of this kinase may be exploited as a novel approach to reinforcing CAR-T therapy against cancer.

## Teaser

Elongation factor-2 kinase is a critical determinant of the fate and antitumor immunity of T killer cells.

## INTRODUCTION

Successful chimeric antigen (Ag) receptor (CAR) T cell therapy for cancer encounters several barriers, including insufficient amounts of tumor Ag-specific T cells because of clonal erasure, poor activation of T cells, accumulation of tolerogenic Ag-presenting cells in the tumor microenvironment (TME), and formation of an immunosuppressive TME (*1*). Mounting evidence has shown that the metabolic status of immune cells and tumor cells can greatly affect antitumor immunity (*2*). Immune activation, acquisition of effector functions, and generation of immune memory are all closely associated with alterations in cellular metabolism. A few connections between metabolic reprogramming and T cell differentiation, survival, and function have been reported recently (*3*–*6*). However, the precise molecular mechanisms and pathways involved remain to be fully elucidated.

Eukaryotic elongation factor-2 kinase (*eEF-2K*), a member of the atypical α-kinase family, is an evolutionarily conserved regulator of protein synthesis. This kinase phosphorylates eEF-2, a 100-kDa protein that promotes ribosomal translocation from the A site to the P site and induces movement of mRNA along the ribosome during translation (*7*). Phosphorylation of eEF-2 on Thr^56^ by *eEF-2K* terminates peptide elongation by decreasing the affinity of this elongation factor for the ribosome. Several studies, including our own (*8*, *9*), have demonstrated that various stress factors such as growth factor deprivation, nutrient deficiency, and oxidative and chemical insults are potent stimulators of *eEF-2K*. Moreover, the activity of this kinase is critically required for survival of stressed cells (*8*, *10*–*15*). We have also reported that *eEF-2K* plays a crucial role in regulating autophagy and cellular adenosine triphosphate (ATP) in tumor cells (*8*, *10*, *11*), and in promoting the Warburg effect. Here, we report our finding that *eEF-2K* has a critical role in determining the fate, function, and antitumor immunity of cytotoxic CD8^+^ T cells (CTLs). Using T cells from *eEF-2K* knockout (KO) mice, we demonstrate that loss of *eEF-2K* significantly reduces the survival and function of CTLs, and this is associated with altered cell proliferation, premature cellular senescence and exhaustion, activated Akt–mammalian target of rapamycin (mTOR)–S6 kinase (S6K) signaling, and reprogrammed metabolism. These findings may have important implications in developing more effective strategies to improve CAR-T therapy for cancer.

## RESULTS

### Loss of eEF-2K alters the fate and function of CD8^+^ T cells

**Fig. 1.**
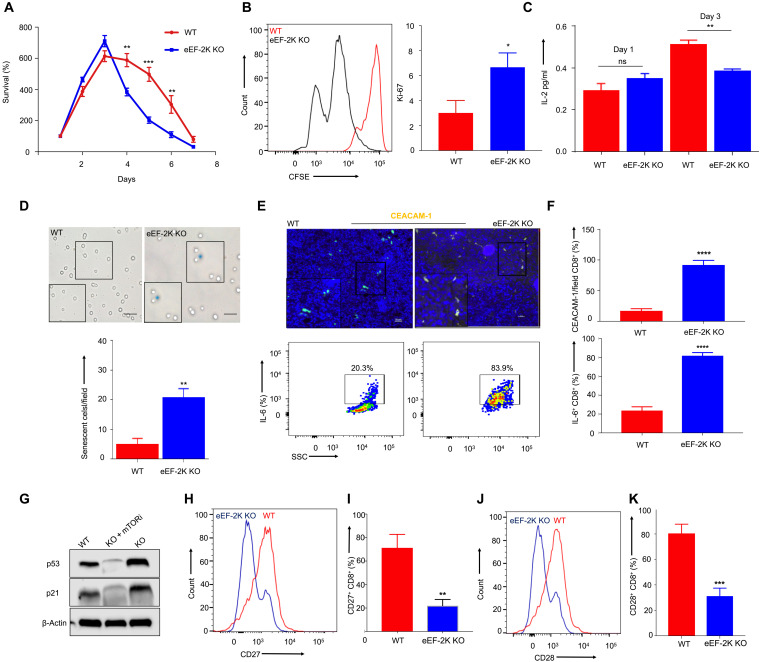
Loss of eEF-2K alters the survival, proliferation, and function of CD8^+^ T cells. CD8^+^ T cells from WT or eEF-2K KO C57BL/6 mice were activated with anti-CD3/CD28 antibodies. The percent survival, cytokine production, and proliferation were assessed. (**A**) Percent survival. (**B**) Proliferation by CFSE-based flow cytometric staining and mass spectrometric proteomics analysis of Ki-67. (**C**) ELISA of IL-2 production on days 1 and 3 after stimulation. (**D**) Comparative SABG staining analysis. The SABG-stained (blue colored) cells were counted, and the mean value from three independent fields was represented graphically. **P < 0.01. High SABG staining and p53 and p21 expression are indicative of senescent phenotype of CD8^+^ T cells. (**E**) CEACAM-1 expression as assessed by confocal microscopy (dual expression of red and green) and IL-6 expression by flow cytometric analysis on WT versus eEF-2K KO CD8 T cells, indicating senescence. ns, not significant. **P < 0.01. (**F**) Graphical representation of CEACAM-1–expressing cells per field and independent replicates of flow cytometric analysis of IL-6 cytokine. (**G**) Western blot analysis of cell cycle arrest senescence-associated p53 and p21 used with mTOR inhibitor rapamycin. (**H**) Flow cytometric histogram analysis of CD27 costimulatory marker. (**I**) Graphical analysis of independent replicates of CD27 expression derived from flow cytometric dot-plot analysis. (**J**) Flow cytometric histogram representation of CD28 costimulatory marker. (**K**) Graphical analysis of independent replicates of CD28 expression derived from flow cytometric dot-plot analysis. Data shown are representative values derived from three identical and independent experiments. *P < 0.05; **P < 0.01; ***P < 0.005; ****P < 0.0001, unpaired *t* test.

To determine the effects of eEF-2K on CD8^+^ T cells, we isolated the T cells from either the wild-type (WT) or eEF-2K KO C57BL/6 mice and then stimulated them with anti-CD3/CD28 antibodies. We found that 3 days after stimulation, the survival of eEF-2K KO CD8^+^ T cells peaked slightly higher than that of WT CD8^+^ T cells but was significantly lower than the controls (twofold change; *P* < 0.05) (Fig. 1A) on days 4, 5, and 6 after stimulation; nevertheless, eEF-2K KO CD8^+^ T cells were more proliferative than WT CD8^+^ T cells after activation, as analyzed by carboxyfluorescein succinimidyl ester (CFSE)–based flow cytometry and by Ki-67 expression (Fig. 1B). Correlatively, significantly decreased production of interleukin-2 (IL-2) was observed in eEF-2K KO CD8^+^ T cells compared to WT CD8^+^ T cells 3 days after their activation (Fig. 1C). In addition, 3 to 4 days after stimulation by anti-CD3/CD28 antibodies, a significantly greater amount of senescence-associated β-galactosidase (SABG)–positive cells were found in the population of eEF-2K KO CD8^+^ T cells than in WT CD8^+^ T cells (*P* < 0.001) (Fig. 1D). This increase in SABG-positive cells was accompanied by elevations of CEACAM-1 (carcinoembryonic Ag-related cell adhesion molecule 1; also known as CD66a) and IL-6 (Fig. 1, E and F) and increased expression of p53 (a cyclin-dependent kinase inhibitor) and p21 (an inducer of cellular senescence) (Fig. 1G), as well as reductions of the costimulatory markers, CD27 (Fig. 1, H and I) and CD28 (Fig. 1, J and K, and fig. S1B), all of which are associated with activation of cellular senescence (*16*, *17*). These results indicate that premature senescence is triggered in CD8^+^ T cells deficient in eEF-2K. Furthermore, as compared with WT CD8^+^ T cells, an increased expression of programmed cell death protein 1 (PD-1) and decreased expression of CD62L were observed in eEF-2K KO CD8^+^ T cells 5 days following their activation (fig. S1A), suggesting that ablation of eEF-2K results in an exhausted state of CD8^+^ T cells. To explore the molecular mechanisms underlying the dysfunctional status of eEF-2K KO CD8^+^ T cells, we performed liquid chromatography–tandem mass spectrometry (LC-MS/MS) analysis and found that 1934 proteins were differentially expressed: 928 proteins in eEF-2K KO CD8^+^ T cells versus 1006 proteins in their WT counterparts; 617 proteins remained unaffected (fig. S2A). Moreover, comparative global proteomics analysis also revealed that the eEF-2K KO CD8^+^ T cells were resistant to apoptosis, a hallmark of senescence, and supported our hypothesis by showing higher expression of senescence markers (fig. S2B). Together, these experiments demonstrated a critical role for eEF-2K in maintaining the robustness and function of CD8^+^ T cells, and indicate that loss of this kinase is detrimental to the functional status of CD8^+^ T cells.

### Cellular metabolism is reprogrammed in CD8^+^ T cells deficient in eEF-2K

**Fig. 2.**
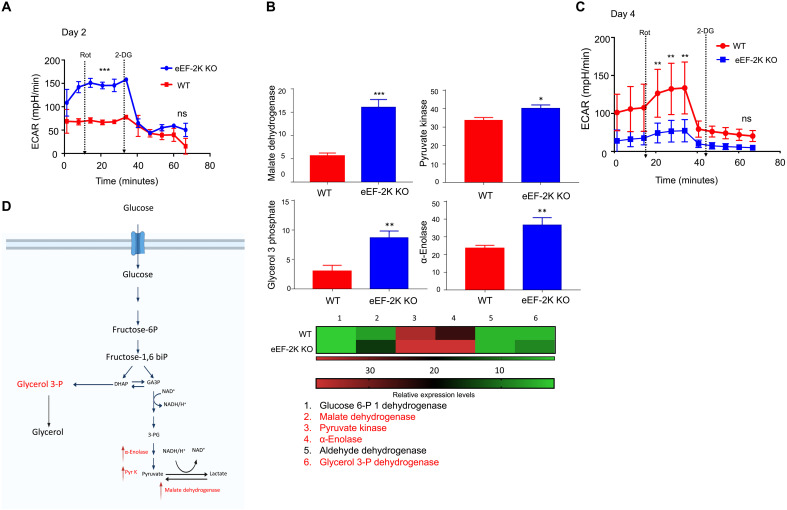
CD8^+^ T cells deficient in eEF-2K are more metabolically active than WT CD8^+^ T cells. CD8^+^ T cells were isolated from WT or eEF-2K KO C57BL/6 mice. The CD8^+^ T cells were subsequently purified and activated with anti-CD3/CD28 antibodies, supplemented with IL-2 cytokine, and grown for 3 to 4 days. The WT or eEF-2K KO CD8^+^ T cells were then analyzed for ECAR using Seahorse XFe96 analysis as per the manufacturer’s protocol. (**A**) Comparative ECAR of eEF-2K KO CD8^+^ T cells compared to that of WT CD8^+^ T cells on day 2 after activation with anti-CD3/CD28 antibodies. (**B**) LC/MS-MS proteomics spectral analysis shown as bar graphs of intermediates of glycolysis pathway from day 3 post-activated CD8^+^ T cells. (**C**) Day 4 post-activation ECAR analysis of WT versus eEF-2K KO CD8^+^ T cells. (**D**) Schematic representation of the glycolytic pathway showing the enzymes up-regulated in CD8^+^ T cells deficient in eEF-2K in red color. Data shown are representative values derived from three independent experiments. *P < 0.05; **P < 0.01; ***P < 0.005, unpaired *t* test.

As metabolic reprogramming is intimately associated with the differentiation, survival, and function of immune cells, and our previous studies showed that expression or activity of eEF-2K has an important regulatory role in production of cellular energy (*8*), we determined whether the impact of eEF-2K on the fate and function of CD8^+^ T cells is mediated through metabolic reprogramming. Seahorse metabolic profiling was performed to analyze cellular metabolism, and the results showed that the activated eEF-2K KO CD8^+^ T cells had a higher basal extracellular acidification rate (ECAR) than the activated WT CD8^+^ T cells (Fig. 2A), indicating that CD8^+^ T cells lacking eEF-2K are more metabolically active; nevertheless, ECAR was decreased considerably 4 days following activation (Fig. 2C). We observed baseline ECAR disruptions in eEF-2K KO CD8^+^ T cell metabolism 4 days after activation, which may be attributable to alterations in metabolic homeostasis and glycolytic activity of these cells. These results suggest that the initial metabolic stress on eEF-2K KO CD8^+^ T cells may lead to their fatigue. To compare the proteome of WT CD8^+^ T cells and eEF-2K KO CD8^+^ T cells, we performed a quantitative global proteomics analysis. This analysis revealed significantly higher production of malate dehydrogenase, pyruvate kinase, α-enolase, aldehyde dehydrogenase, and glycerol 3-phosphate in eEF-2K KO CD8^+^ T cells than in control cells on day 3 after activation (Fig. 2B), further suggesting a high glycolytic activity and metabolic exhaustion in eEF-2K KO CD8^+^ T cells (Fig. 2D). Thus, the metabolic reprogramming observed in eEF-2K KO CD8^+^ T cells may account for their altered survival, proliferation, and functional status (Fig. 1).

### Akt-mTOR-S6K signaling is hyperregulated in CD8^+^ T cells lacking eEF-2K

**Fig. 3.**
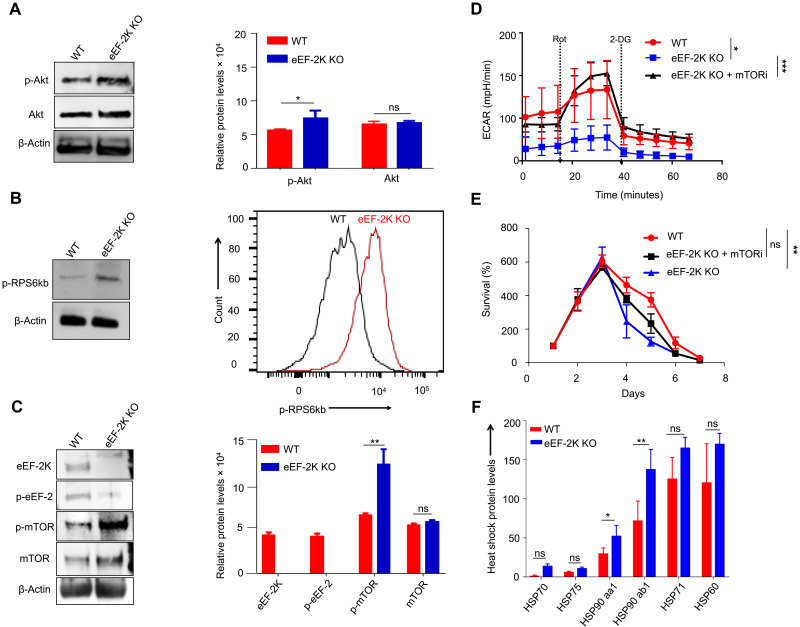
Loss of eEF-2K up-regulates the Akt-mTOR signaling and HSP90 in CD8^+^ T cells. (**A**) Western blot analysis of up-regulation of Akt–phospho-Akt proteins followed by quantification using ImageJ software of independent experiments. (**B**) Phospho-RPS6kb was determined by Western blots and validated by phospho-flow cytometric analysis. (**C**) Western blot analysis confirms that loss of eEF-2K up-regulates the mTOR signaling pathway. Independent blots were quantified with ImageJ software and normalized to β-actin. (**D**) Seahorse metabolic profile shows that the dysfunctional metabolism in eEF-2K KO CD8^+^ T cells is partially restored to WT levels on treatment with rapamycin. (**E**) Survival of eEF-2K KO CD8^+^ T cells was partially restored to WT levels on treatment with rapamycin. (**F**) The LC-MS/MS spectra indicated that all major heat shock proteins (HSPs) were up-regulated in eEF-2K KO CD8^+^ T cells compared to their WT counterparts with significant up-regulation of HSP90-aa1 and HSP90-ab1. mTORi = rapamycin. Data shown are representative values derived from three independent experiments. *P < 0.05; **P < 0.01; ***P < 0.005, unpaired *t* test.

It has been reported that Akt-mTORC-S6K signaling has a critical role in T cell differentiation (*18*). To determine how cellular metabolism is reprogrammed in CD8^+^ T cells lacking eEF-2K, using eEF-2K KO CD8^+^ T cells and WT CD8^+^ T cells, we compared the activity of the Akt-mTOR-S6K signaling, which is a central pathway in cellular metabolism, proliferation, growth, and survival (*19*). We found that phosphorylation of Akt, mTOR, and S6K was increased in eEF-2K KO CD8^+^ T cells compared to WT CD8^+^ T cells (Fig. 3, A to C), suggesting that depletion of eEF-2K causes hyperactivation of the Akt-mTOR-S6K signaling, and this may be responsible for the metabolic alteration observed in CD8^+^ T cells deficient in eEF-2K (Fig. 2). To further demonstrate the role of the higher Akt-mTOR-S6K activity in the metabolic reprogramming that occurred in eEF-2K KO CD8^+^ T cells, we treated these cells with rapamycin, a selective inhibitor of mTOR, and then analyzed ECAR. Figure 3D shows that on day 4 after activation, the metabolism of the rapamycin-treated eEF-2K KO CD8^+^ T cells was substantially different from that of the untreated cells but was similar to that of WT CD8^+^ T cells; this observation supported the role of the Akt-mTOR-S6K signaling in controlling CD8^+^ T cell metabolism. In addition, the survival of eEF-2K KO CD8^+^ T cells was partially improved (Fig. 3E). The expression of PD-1 and Tim-3, the proteins known to regulate immune surveillance of cancer cells (*20*), was also partially restored in the presence of rapamycin (fig. S3) on day 6 after activation of these cells. The differential PD-1 expression in WT CD8^+^ T cells, as shown in figs. S1A and S3, may be attributable to the differential activation status or different time points of data collection after activation. These data suggest that the altered activity of the Akt-mTOR-S6K signaling in eEF-2K KO CD8^+^ T cells may play a critical role in modulating the fate and function of CD8^+^ T cells.

**Fig. 4.**
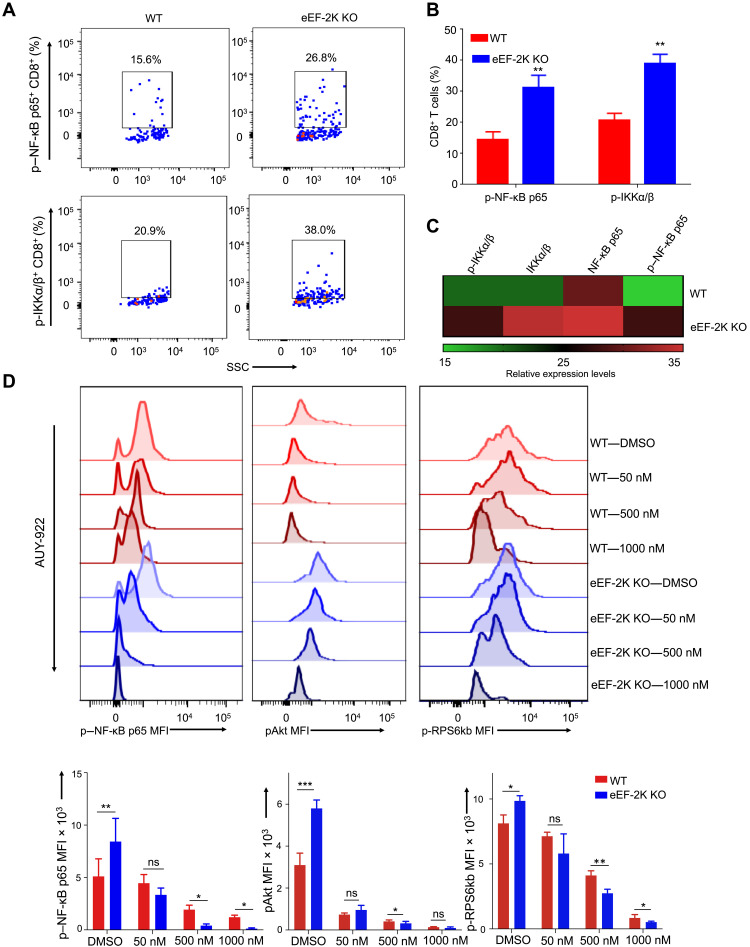
Involvement of NF-κB in the up-regulation of Akt-mTOR signaling in CD8^+^ T cells deficient in eEF-2K. Cell lysates from WT or eEF-2K KO CD8^+^ T cells on days 1 and 3 after activation were analyzed for NF-κB activity by flow cytometry. (**A**) NF-κB p65 and phospho-IKKα/β were analyzed and compared by flow cytometric analyses for WT versus eEF-2K KO CD8^+^ T cells. (**B** and **C**) Graphical and heatmap representation of triplicates of flow cytometric dot-plot analysis of simultaneous increase in phospho–NF-κB p65 from day 3 protein lysates. (**D**) Increasing concentration of HSP90 inhibitor (AUY-922) was administered to day 2 cultures of WT versus eEF-2K KO CD8^+^ T cells, and the cells were analyzed for the expression of phospho–NF-κB p65, phospho-Akt, and phospho-RPS6kb on day 3 after activation. Heatmap analysis of triplicates was taken from flow cytometric dot-plot analysis. The flow cytometric plots are representative values shown from three independent experiments. Graphical data shown are means ± SD from values derived from three independent experiments. **P < 0.01, unpaired *t* test.

Our LC-MS/MS analysis also revealed that among the differentially expressed proteins, heat shock protein 90 (HSP90) was expressed at substantially higher levels in eEF-2K KO CD8^+^ T cells than in WT CD8^+^ T cells (Fig. 3F). This finding is consistent with a previous study showing that the amounts of this chaperone protein were increased in the eEF-2K–depleted human cells and eEF-2K KO mouse embryonic fibroblasts (*21*). As the nuclear factor κB (NF-κB) pathway is controlled by HSP90 (*22*, *23*) and can modulate the activity of Akt and mTOR (*24*, *25*), we next analyzed the NF-κB pathway in CD8^+^ T cells with or without ablation of eEF-2K. Phosphorylation of NF-κB p65 and IKKα/β (inhibitor of NF-κB kinase α/β) subunits is a critical determinant of the activity of NF-κB pathway (*26*). We found that on day 3 after activation, the up-regulation of HSP90 in eEF-2K KO CD8^+^ T cells was accompanied by increases of phospho–NF-κB p65 and phospho-IKKα/β (Fig. 4, A to C). Treatment of the CD8^+^ T cells with AUY-922, a small-molecule inhibitor of HSP90, caused dose-dependent reductions of phospho–NF-κB p65, phospho-Akt, and phospho-ribosomal protein S6 kinase B1 (RPS6kb) (Fig. 4D). These results suggest that the up-regulation of HSP90/NF-κB induced by eEF-2K ablation may be responsible for the activation of Akt-mTOR-S6kb signaling, leading to an early enhanced metabolic state of eEF-2K KO CD8^+^ T cells.

### Impact of eEF-2K expression on antitumor efficacy of CAR-T therapy

**Fig. 5.**
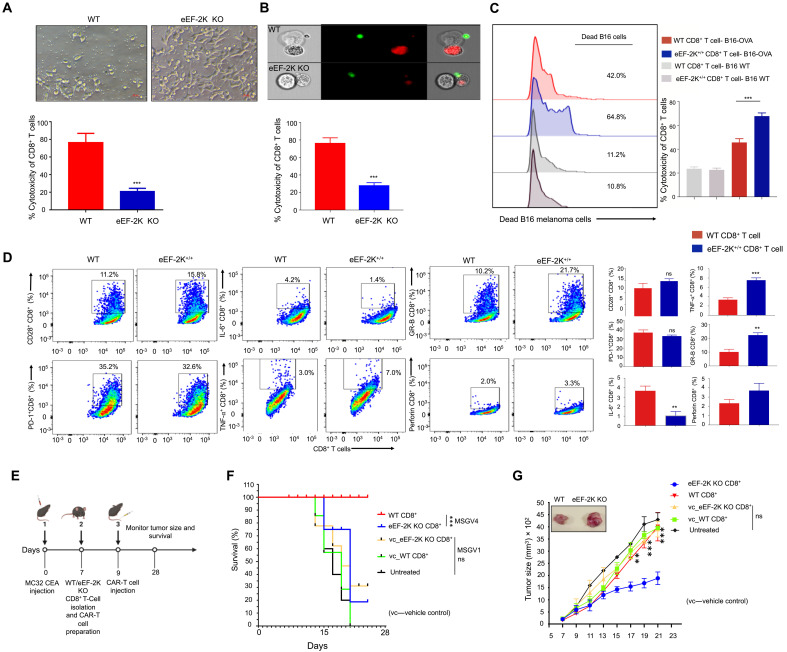
Loss of eEF-2K in CAR-CD8^+^ T cells diminishes their cytocidal activity against tumor cells, while overexpressing eEF-2K increases the cytocidal activity of CD8^+^ T cells. MC32 CEA colon cancer cells were cocultured with murine WT and eEF-2K KO CD8^+^ CEA CAR-T cells for 6 hours. (**A**) Bright-field images of MC32 CEA colon cancer cells cocultured with the murine WT or eEF-2K KO CD8^+^ CEA CAR-T cells. The % of live cells were counted per field at *T* = 0 hours and then at *T* = 6 hours to quantify the live-dead ratio and expressed graphically. (**B**) Image-stream analysis of murine WT and eEF-2K KO CD8^+^ T cells showing that the CD8^+^ T cells attach to MC32 CEA colon cancer cells, resulting in the death of these cancer cells. (**C**) B16 or B16-OVA melanoma cells were cocultured with the murine OT-I WT or eEF-2K–overexpressing CD8^+^ T cells for 6 hours. The half-offset histogram plot shows the killing potential of eEF-2K–overexpressing CD8^+^ T cells against B16-OVA cells. The dead-live % of tumor cells was analyzed. (**D**) The flow cytometric dot plots represent the functional profile of OT-I WT and eEF-2K–overexpressing CD8^+^ T cells taken from their coculture with B16-OVA melanoma cells. (**E**) Schematic of adoptive immunotherapy experiment. (**F**) Survival of tumor-bearing mice in different groups. (**G**) Growth curves of MC32 CEA murine tumor. Graphical data shown are means ± SD from values derived from three independent experiments. **P < 0.01, unpaired *t* test.

To determine the importance of eEF-2K in the CD8^+^ T cell–mediated antitumor immunity, we isolated CD8^+^ T cells from the spleen and lymph nodes of WT or eEF-2K KO mice and transduced them with a chimeric carcinoembryonic antigen (CEA) receptor construct to generate the CEA-Ag–specific CD8^+^ T cells. Then, we cocultured the WT CEA-specific CD8^+^ T cells or the CEA-specific eEF-2K KO CD8^+^ T cells with MC32 murine colon carcinoma cells expressing CEA. The cytocidal activity of CD8^+^ T cell was assessed using microscopy observation (Fig. 5A), image cytometry analyses (Fig. 5B), and lactate dehydrogenase (LDH) release assays (fig. S4B). The cytocidal activity of the CEA-specific eEF-2K KO CD8^+^ T cells was significantly lower than that of the WT CEA-specific CD8^+^ T cells (*P* = 0.0009 and *P* = 0.0002, respectively) (Fig. 5, A and B), indicating that the cytotoxicity of eEF-2K KO CEA-Ag–specific CD8^+^ T cells is significantly compromised compared to WT CEA-Ag–specific CD8^+^ T cells. To examine the possible off-target effects of these CAR-T cells, we cocultured the CEA-specific CD8^+^ T cells with MC38 colon carcinoma cells not expressing CEA (*27*) and then performed identical assays as above. No off-target effects were observed in these assays (fig. S4A).

To further demonstrate the effect of eEF-2K on cytocidal activity of CD8^+^ T cells, we isolated CD8^+^ T cells from OT-I T cell receptor (TCR) transgenic mice, transduced these cells retrovirally with an eEF-2K expression vector, and determined the effects of the overexpression of eEF-2K on the cytotoxicity and function of the CD8^+^ T cells. These OT-I CD8^+^ T cells recognize the ovalbumin (OVA) expressed on B16-OVA melanoma cells. We showed that the cytotoxicity of the eEF-2K^+/+^ CD8^+^ T cells overexpressing eEF-2K was significantly higher than that of their WT counterparts (*P* = 0.0008) (Fig. 5C and fig. S4C). Off-target effects were ruled out by coculturing WT and eEF-2K^+/+^ CD8^+^ OT-I T cells with control B16 (OVA^null^) melanoma cell lines (Fig. 5C). Overexpression of eEF-2K in OT-I T cells (eEF-2K^+/+^) also improved their functional profile, as evidenced by the increased expression of CD28 and reduced expression of PD-1. In addition, some anti-inflammatory and prosurvival cytokines such as tumor necrosis factor–α (TNF-α), interferon-γ (IFN-γ), IL-2, and IL-6 were all improved in the eEF-2K^+/+^ CD8^+^ T cells (Fig. 5D and fig. S6B). These results imply that the compromised cytotoxicity of CD8^+^ T cells can be recovered by overexpressing eEF-2K in those T cells, irrespective of the Ag specificity of the TCR.

To recapitulate the in vitro observations in animal tumor model, we inoculated mice with MC32-CEA tumor cells (1 × 10^6^ cells per mouse) subcutaneously in the right lateral flank, followed by intravenous injection of WT or eEF-2K KO CEA-specific CAR-T cells (5 × 10^6^ cells per mouse) (Fig. 5E). In this tumor model, we observed that the tumoricidal effect of WT CAR-T cells was substantially stronger than that of eEF-2K KO CAR-T cells (Fig. 5, F and G). All the mice receiving an intravenous infusion of WT CEA CD8^+^ CAR-T cells survived at least 28 days after tumor induction, whereas the survival of the mice treated with eEF-2K KO CAR-T cells declined rapidly from day 15 onward (Fig. 5F). In addition, the tumor-inhibitory effect of WT CEA CD8^+^ CAR-T cells was significantly weakened when eEF-2K was ablated (Fig. 5G). These results demonstrate that eEF-2K is crucial for the antitumor activity of CD8^+^ T cells.

### CD8^+^ CAR-T cells deficient in eEF-2K show a reduced ability to infiltrate the TME

**Fig. 6.**
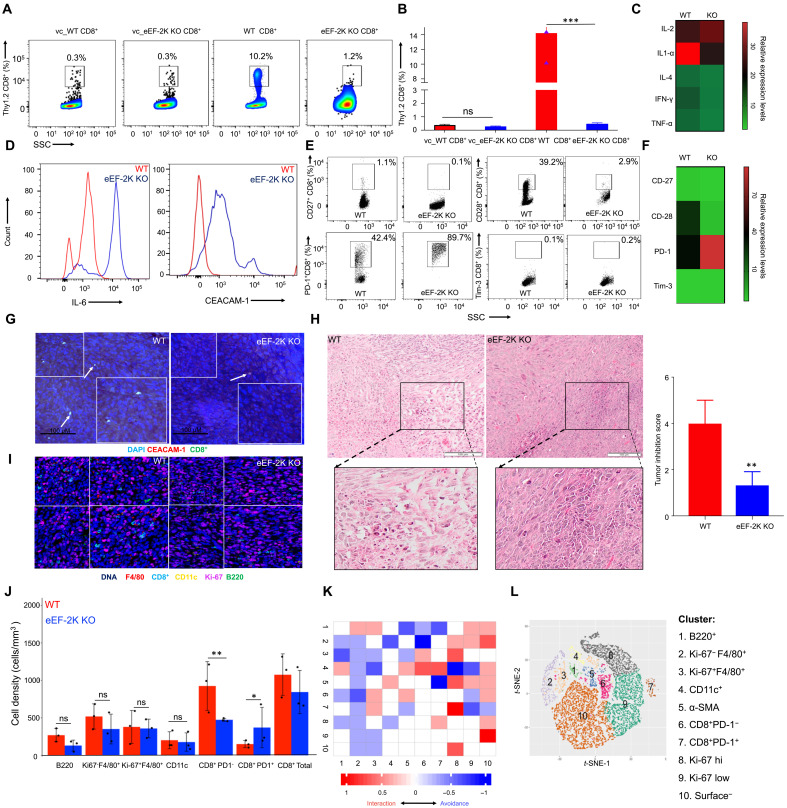
Loss of eEF-2K in CAR-CD8^+^ T cells impairs their ability to infiltrate tumor tissues. On day 28 after induction of tumors, the explanted tumor was analyzed. (**A**) TILs were gated for Thy1.2^+^CD8^+^ T cells and enumerated by flow cytometric analysis. Data shown are means ± SD from three independent experiments (*n* = 5). ***P < 0.005, unpaired *t* test. (**B**) Graphical representation of TILs derived from flow cytometric analysis. (**C**) Heatmap analysis of TNF-α, IFN-γ, IL-4, IL1-α, and IL-2 in tumor-infiltrating and anti-CD3/CD28 activated CD8^+^ T cells. (**D**) IL-6 and CEACAM-1 expression of TILs by flow cytometric analysis. (**E**) Expressions of CD27, CD28, PD-1, and Tim-3 on TILs by flow cytometric analysis. (**F**) Heatmap analysis of CD27, CD28, PD-1, and Tim-3. (**G**) Confocal microscopy detection of CD8^+^ CAR-T cells in MC32CEA tumors. The infiltrating CD8^+^ T cells were enumerated from five different fields and graphically plotted. (**H**) H&E staining of tumor sections. (**I**) Assessment of the TME with overlaid images of MC32 CEA tumor with five markers. (**J**) Quantification of different immune cell populations. (**K**) Differential neighborhood analysis of cell clusters of the two adoptively transferred groups represented by a single subtractive heatmap showing the unique associations of eEF-2K KO CD8^+^ T cells subtracted from the WT CD8^+^ T cells. The rows represent the cell phenotypes of interest, whereas the columns represent the cell phenotypes in neighborhood. (**L**) viSNE dimension reduction algorithm plots of WT versus eEF-2K KO CD8^+^ T cells. *P < 0.05 and **P < 0.01; unpaired *t* test.

Furthermore, we examined the tumor infiltration ability of the injected CAR-T cells using flow cytometric analysis of the explanted tumors. Much fewer CEA-specific eEF-2K KO CAR-T cells were detected in the tumor tissues as compared with control cells (Fig. 6, A and B). CEA-specific eEF-2K KO CAR-T cells had a significantly lower penetration into the TME, which was similar to the non-CEA control CD8^+^ T cells. In addition, the heatmap constructed from the flow cytometric dot plots exhibited a dysfunctional state of the tumor-infiltrating eEF-2K KO CAR-T cells: Compared to WT CD8^+^ CAR-T cells, the CD8^+^ CAR-T cells lacking eEF-2K produced significantly less inflammatory cytokines TNF-α, IFN-γ, and IL-4, and the prosurvival cytokine IL-1α, but an equivalent amount of IL-2 cytokine (Fig. 6C and fig. S5, E and I). In addition, the CEA-specific eEF-2K KO CAR-T cells displayed a compromised activation and high level of exhaustion, as the eEF-2K KO CAR-T cells infiltrating the TME produced more intracellular IL-6 cytokine and CEACAM-1 on their surfaces (Fig. 6D), significantly lower expression of CD27 and CD28, but significantly higher expression of PD-1 and Tim-3 (Fig. 6, E and F, and fig. S5, A and D) than the WT CAR-T cells. Confocal microscopic analysis confirmed the lower penetration of eEF-2K KO CAR-T cells than WT control cells (Fig. 6G and fig. S5J). The hematoxylin and eosin (H&E) staining of the tumor specimens showed that there were considerably greater amounts of tumor cells in the xenografts from the mice treated with the CEA-specific eEF-2K KO CAR-T cells than in the xenografts from the mice receiving the WT CEA CD8^+^ CAR-T cells (Fig. 6H). These results indicate that loss of eEF-2K not only impairs the tumoricidal activity of the CEA-specific CAR-T cells but also weakens their ability to infiltrate tumor tissues. Furthermore, we analyzed and compared the functional profile of the tumor-infiltrating CAR-T cells with or without depletion of eEF-2K. Mass cytometric analysis of the explanted tumors validated that total penetration of the WT CEA CD8^+^ T cells into TME was higher than that of CEA CAR CD8^+^ T cells with ablation of eEF-2K (Fig. 6, I and J). In addition, the number of CD8^+^ PD-1^+^ T cells was significantly higher in the population of CEA CAR CD8^+^ T cells lacking eEF-2K, whereas the number of the CD8^+^ PD-1^−^ T cells was significantly higher in the population of the WT CEA CAR CD8^+^ T cells (*P* = 0.1372; Fig. 6J), suggesting that exhaustion and dysfunction may account for the low penetration of CAR CD8^+^ T cells deficient in eEF-2K. In addition, in the tumor specimens from the mice receiving the WT CEA CAR CD8^+^ T cells, an increase in Ki-67^−^ macrophages (Ki-67^−^F4/80^+^) was detected, whereas the tumor specimens from the mice receiving eEF-2K KO CAR-T cells had high amounts of Ki-67^+^ macrophages (Ki-67^+^F4/80^+^) (Fig. 6, I and J), another difference in the antitumor efficacy between the WT CEA CAR-T cells and eEF-2K KO CEA-specific CAR-T cells. The immune cells in TME were also compared using neighborhood joining heatmaps and *t*-distributed stochastic neighbor embedding (*t*-SNE) plots (Fig. 6, K and L). The representative subtractive heatmap shows that the TME of the mice receiving eEF-2K KO CAR-T cells may be more favorable for proliferation of CD8^+^PD-1^+^ T cells, as these cells can preferably interact with Ki-67 protein. On the other hand, CD8^+^PD-1^−^ T cells, which are functionally active and less exhausted, are less likely to interact with Ki-67 protein. The functional CD8^+^PD-1^−^ T cells display a much lower potential of interacting with α-smooth muscle actin (α-SMA) protein, suggesting a reduced efficacy of killing tumor cells (Fig. 6K). The *t*-SNE plot generated from the heatmap shows an overview of the TME of the mice receiving an infusion of CAR-T cells (Fig. 6L). All these results consistently demonstrate a crucial role of eEF-2K in controlling the persistence and function of CTLs.

## DISCUSSION

CTLs are a key component of antitumor immunity, yet the critical determinants of their function and fate remain to be fully defined. Here, we report a previously unappreciated role of eEF-2K in sustaining the survival and cytocidal activity of CTLs. The impetus for this study is our finding that eEF-2K has an important role in regulating stress responses and cellular metabolism (*4*–*8*), and the importance of metabolic reprogramming in controlling the survival, differentiation, expansion, and activation of immune cells. We demonstrate that eEF-2K is essential for maintaining the robustness and function of CD8^+^ T cells, and that loss of this kinase is detrimental to their functional status and fate (Fig. 1). We found that eEF-2K is a critical determinant of antitumor immunity of CD8^+^ T cells, as their cytocidal activity is reduced significantly both in vitro (Fig. 5, A and B) and in vivo (Fig. 5, F and G) when eEF-2K is depleted. In addition, the ability to penetrate tumors and tumor cell–killing functions are impaired in CD8^+^ T cells deficient in eEF-2K (Fig. 6A), which could be a consequence of premature exhaustion and senescence of these cells (Fig. 1). We further show that cellular metabolism is altered in the eEF-2K–deficient CD8^+^ T cells (Fig. 2), which is associated with hyperactivation of the Akt-mTOR-S6K pathway (Fig. 3). Thus, the metabolic reprogramming mediated by eEF-2K may account for the effects of eEF-2K on CD8^+^ T cells.

As protein synthesis is one of the most notable consumers of cellular energy and eEF-2K is a key regulator of protein synthesis and a critical checkpoint in energy consumption (*9*), deficiency of this kinase may lead to metabolic catastrophe. Therefore, the alterations of cell proliferation, survival, senescence, and function (Fig. 1) as well as metabolic activity (Fig. 2) observed in eEF-2K KO CD8^+^ T cells could be a cellular response to metabolic stress, which is critical for the sustained activity and survival of CD8^+^ T cells (*28*–*30*). Nevertheless, excessive stress responses can negatively affect cell fate, as evidenced by premature senescence, shorter life span, and weakened function of CD8^+^ T cells deficient in eEF-2K (Fig. 1). The premature and dysfunctional state of eEF-2K KO CD8^+^ T cells (Fig. 1), which likely results from the metabolic reprogramming following activation, significantly weakens their cytocidal activity (Fig. 5, A and B), tumoricidal action (Fig. 5, F and G), and tumor infiltration ability (Fig. 6A). Our experiments could differentiate the tumor-infiltrating lymphocytes (TILs) from the tissue-resident CD8^+^ T cells but were barely able to distinguish the infiltration and expansion of these cells within the TME. The impact of eEF-2K expression on the cytocidal activity of CD8^+^ T cells was further validated by the experiments demonstrating that augmenting eEF-2K in tumor Ag-specific CD8^+^ T cells enhanced the antitumor efficacy of CTLs and improved their functionality (Fig. 5, C and D).

The altered metabolic status of eEF-2K KO CD8^+^ T cells was manifested by higher glycolytic activity (Fig. 2) and up-regulated Akt-mTOR signaling following activation (Fig. 3, A to C). The enhanced Akt-mTOR activity in eEF-2K KO CD8^+^ T cells is likely caused by HSP90 up-regulation (Fig. 3F) and NF-κB activation (Fig. 4A), as inhibition of HSP90 by AUY-922, a small-molecule inhibitor of HSP90, is accompanied by down-regulation of the Akt-mTOR signaling (Fig. 4D). These results are consistent with a recent report showing that the expression of the metabolic signaling proteins, including NF-κB, mTOR, and pRPS6kb, was up-regulated in the early activated CD8^+^ T cells as a way of metabolic adaptation (*31*). Recently, others have also reported that maintaining the equilibrium of the Akt-mTOR signaling pathway is critical for promoting T cell quiescence, longevity, and homeostasis (*32*). In addition, elevations of HSP90 and the phospho-Akt proteins have been reported in other types of cells deficient in eEF-2K, including the intestinal stem cells, epithelial cells (*33*), human tumor cells, and mouse embryonic fibroblasts (*21*). Similarly, we found that the up-regulations of this signaling also occur in CD8^+^ T cells subjected to eEF-2K ablation (Fig. 3, A and F). It would be interesting to determine whether the activities of that signaling in other subsets of T cells or immune cells are also affected by eEF-2K expression. In addition, previous studies have shown that Akt is involved in the control of the metabolic fate of CD8^+^ T cells via TCR signaling (*34*) and that mTOR activity is up-regulated in the highly proliferative effector CD8^+^ T cells. However, whether eEF-2K expression or activity is lost or reduced in those CD8^+^ T cells remains to be investigated.

**Fig. 7.**
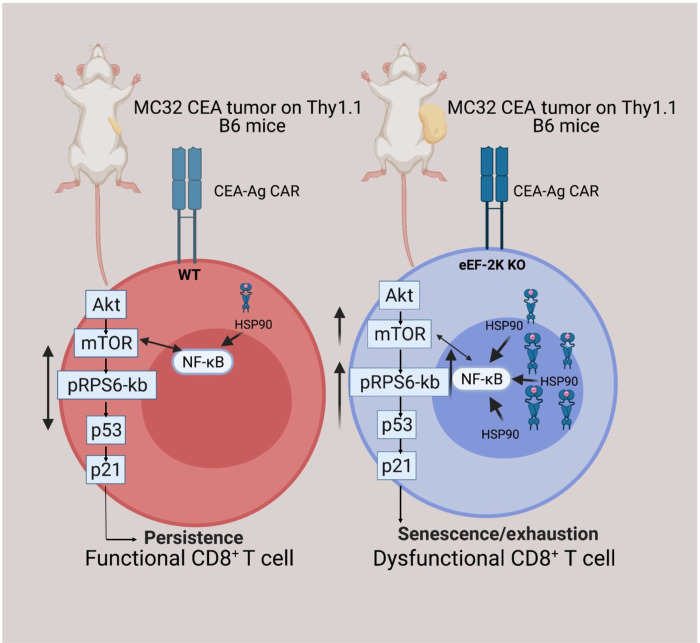
The effects of eEF-2K on CD8^+^ T cells are mediated through the Akt-mTOR-S6K pathway. The illustration shows that Akt-mTOR-S6K signaling is hyperregulated in CD8^+^ T cells lacking eEF-2K. The up-regulation of HSP90/NF-κB induced by eEF-2K ablation may be responsible for the activation of Akt-mTOR-S6kb signaling, leading to an early enhanced metabolic state of eEF-2K KO CD8^+^ T cells.

In summary, this study identifies eEF-2K as a crucial regulator of the antitumor immunity of CTLs. eEF-2K is essential for the viability and function of those CD8^+^ T cells, and the effects of this kinase on these T cells are mediated through the Akt-mTOR-S6K pathway (Fig. 7). Furthermore, we demonstrate that the regulation of CD8^+^ T cells by eEF-2K significantly affects their antitumor function and ability to penetrate the TME. Thus, the critical role of eEF-2K in upholding the activity and function of CTLs warrants further investigation to assess whether therapeutic augmentation of this kinase can be exploited as a novel approach to reinforcing CAR-T therapy against cancer.

## MATERIALS AND METHODS

### Cell lines and culture

Murine colon adenocarcinoma cells (MC38, CEA^−^) or MC32 (MC32, CEA^+^) were grown in Dulbecco’s modified Eagle’s medium (DMEM) with 10% fetal bovine serum (FBS), 1% l-glutamine, and 1% penicillin-streptomycin (*27*). The cells were grown to confluence in 5% CO_2_ incubators and used for in vitro murine CD8^+^ T cell coculture and in vivo solid tumor induction experiments. B16 or B16-OVA melanoma cells were also grown in DMEM with 10% FBS, 1% l-glutamine, and 1% penicillin-streptomycin and maintained in 5% CO_2_ incubators.

### Global proteomics analysis

The sample preparation for LC-MS/MS analysis was performed by using a previously described in-gel digestion methodology for sample preparation from WT and eEF-2K KO CD8^+^ T cells cultured for 3 days after activation with anti-CD3/CD28 antibodies (*35*), with minor modifications. The spectral analysis was performed by extracting the tandem mass spectra, and all MS/MS samples were analyzed using Mascot (Matrix Science, London, UK; version 2.7.0) and X Tandem (2010.12.01.1). Tandem was set up to search a reverse concatenated subset of the contaminants_20120713_UniProt_Mouse_20161004 database with 21,478 entries. Tandem was searched with a fragment ion mass tolerance of 0.80 Da and a parent ion tolerance of 20 parts per million. Carbamidomethyl of cysteine was specified in Mascot and Tandem as a fixed modification. Deamidation of asparagine and glutamine, oxidation of methionine, acetyl of the N terminus, and phosphorylation of serine, threonine, and tyrosine were specified in Mascot as variable modifications. The total differentially expressed proteins in WT versus eEF-2K KO CD8^+^ T cells were enumerated from mass spectrometric analysis results and plotted in a Venn diagram. Comparative protein amounts of Ki-67, malate dehydrogenase, pyruvate kinase, glycerol-3-phosphate, α-enolase, apoptotic and senescent marker proteins, as well as HSPs were enumerated from the data and plotted graphically.

### Animal experiments

eEF-2K KO mice (C57BL/6 background; Thy1.2^+^) were generated previously (*36*). C57BL/6 congenic mice (B6 Thy1.2; Thy1.1^+^) were obtained from The Jackson Laboratory (Bar Harbor, ME) and maintained in-house in specific pathogen–free (SPF) biosafety level 2 (BSL2) facility with 12:12-hour light-dark cycle. Naive CD8^+^ T cells were isolated from the pooled splenocytes and lymph nodes of the WT and eEF-2K KO mice using the negative CD8^+^ T cell selection with a MojoSort Mouse T cell isolation kit (BioLegend, San Diego, CA). The CD8^+^ T cells harvested were used for both in vitro and in vivo adoptive immunotherapy experiments.

C57BL/6 congenic mice (6 to 8 weeks old, male/female) were used for adoptive tumor immunotherapy experiments. The mice were divided into four different groups (*n* = 5) and maintained in SPF-BSL2 facility at 12:12-hour light-dark cycle, 68° to 72°F ambient temperature, and 30 to 70% humidity for tumor immunotherapy experiments.

OT-I TCR transgenic mice (6 to 8 weeks old) were used to isolate CD8^+^ T cells for overexpression of eEF-2K. These mice contain transgenic inserts for mouse Tcra-V2 and Tcrb-V5 genes (*37*) in CD8^+^ T cells. The OVA-specific TCRs on CD8^+^ T cells recognize major histocompatibility complex (MHC) class I–restricted OVA epitope on B16-OVA melanoma cells. The WT or eEF-2K–overexpressing OT-I CD8^+^ T cells were used in in vitro coculture assay with B16-OVA melanoma cells. All animal studies were conducted in accordance with the guidelines of Institutional Animal Care and Use Committee (IACUC no. 2018-0065), Texas A&M University.

### Overexpression of eEF-2K in OT-I CD8^+^ T cells

eEF-2K gene was cloned from pCDNA3-HA-eEF2K (Addgene no. 110160) vector and inserted into the gamma-retroviral vector pMIG modified from the backbone pMSCV 2.2. The pMIG vector was first transduced into platinum-E (Plat-E) retroviral packaging cell line, allowing retroviral packaging with a single plasmid transfection. The viral supernatants were then used to transduce the OT-I CD8^+^ T cells to generate eEF-2K–overexpressing T cells (eEF-2K^+/+^). A mock transduction with empty vector was also performed as a control.

### CAR-T cell preparation

The murine stem cell–based gamma-retroviral vector MSGV1, which was used as a control vector here, is composed of CAR elements of CD28 and CD3z moieties but lacks CEA-Ag expression (*38*). MSGV4 retroviral vector was modified from the MSGV1 background to express CEA-Ag–specific single-chain variable fragment (scFv) with other CAR elements derived from MSGV1 backbone. WT and eEF-2K KO CD8^+^ T cells were transduced with the viral supernatants containing MSGV1 (control; no CEA) or MSGV4 (CEA) (*27*). Briefly, naive CD8^+^ T cells isolated from WT and eEF-2K KO B6 Thy1.2 mice were stimulated and maintained in RPMI 1640 medium [10% FBS, 50 μM 2-mercaptoethanol, 1% penicillin-streptomycin, 1% Non essential amino acids (NEAA), 1% sodium pyruvate, and IL-2 (50 U/ml)].

Retroviral supernatants produced from MSGV1-null or MSGV4-CEA–transduced Plat-E packaging cell line were added to the isolated WT and eEF-2K KO CD8^+^ T cells in RPMI 1640 medium supplemented with polybrene (5 μg/ml; Sigma-Aldrich, St. Louis, MO). The cells were then centrifuged at 32°C for 1 hour and further incubated at the same temperature in 5% CO_2_ incubators for 6 hours. The transduced CD8^+^ T cells were identified by analyzing c-myc expression in a BD Fortessa X-20 flow cytometer (BD Biosciences, San Jose, CA).

### Assays for survival, proliferation, and IL-2 production of CD8^+^ T cells

Naive WT and eEF-2K KO CD8^+^ T cells isolated from B6 Thy1.2 mice were activated by anti-mouse CD3 antibody (clone 2C11; BioLegend, San Diego, CA)/anti-mouse CD28 antibody (clone 37.51; BioLegend, San Diego, CA) and monitored for their survival by trypan blue cell exclusion method using a TC20 automated cell counter (Bio-Rad, USA). The live CD8^+^ T cells were counted and plotted graphically with GraphPad Prism 9. CD8^+^ T cell proliferation was measured by CFSE (Invitrogen, Carlsbad, CA) assay as described previously (*2*, *30*). WT and eEF-2K KO CD8^+^ T cell IL-2 secretion was assessed from day 1 to 3 cell culture supernatants using enzyme-linked immunosorbent assay (ELISA) kits (BioLegend, San Diego, CA) as per the manufacturer’s instructions.

### Western blots

WT and eEF-2K KO CD8^+^ T cells were lysed with radioimmunoprecipitation assay (RIPA) lysis buffer, and 30 μg of protein lysate was tested for SDS–polyacrylamide gel electrophoresis (SDS-PAGE), as described previously with minor modifications (*39*). Briefly, proteins resolved by 10% SDS-PAGE gel were transferred onto polyvinylidene difluoride (PVDF) membranes with a semi-dry electroblotting system. The PVDF membranes were then blocked for 1 hour at room temperature in 2% bovine serum albumin and subsequently probed with primary antibodies for eEF-2K (catalog no. 3692, Cell Signaling Technology, Danvers, MA), phospho-Akt (S472, clone no. 104A282, BD Biosciences, San Jose, CA), Akt (clone no. 094E10, BioLegend, San Diego, CA), phospho-mTOR (S2448, catalog no. 2971, Cell Signaling Technology, Danvers, MA), mTOR (catalog no. A301-143A, Bethyl Lab, Montgomery, TX), and phospho-RPS6kb (S235/S236, clone no. A17020B, BioLegend, San Diego, CA). The membranes were then washed and probed with appropriate horseradish peroxidase–conjugated secondary antibodies (Cell Signaling Technology, Danvers, MA) as required. The blots were stripped and reprobed with β-actin antibody, which served as the loading control.

### Assay for SABG activity

WT and eEF-2K KO CD8^+^ murine T cells were isolated from WT and eEF-2K KO B6 Thy1.2 mice and cultured in RPMI 1640 medium as described previously, until 8 days after activation with anti-CD3/CD28 antibodies. The senescence of WT CD8^+^ T cells or eEF-2K KO CD8^+^ T cells was then compared by performing SABG staining (no. CBA-230, Cell Biolabs, San Diego, CA) (*40*, *41*) as per the manufacturer’s protocols. SABG-positive senescent cells stain blue-green in the assay (*42*). The senescent CD8^+^ T cells were imaged and quantified with a Leica Slide Scanner microscope.

### Adoptive cell transfer

C57BL/6 congenic mice were subcutaneously injected with 1 × 10^6^ MC32 CEA tumor cells in the right lateral flank on day 0. Following tumor injection, the mice were divided into five different groups. CD8^+^ T cells were isolated from WT and eEF-2K KO B6 Thy1.2 mice on day 5 and retrovirally transduced with either MSGV4 (CEA) or MSGV1 CAR constructs in a manner described previously (*27*). T cells were cultured for two more days after transduction and subsequently intravenously infused into different tumor-bearing mice (Fig. 5E). Untreated tumor-bearing mice did not receive any infusion of CD8^+^ T cells and served as the control group. The mice were monitored for survival and tumor size up to day 28 after tumor induction. The experiment was terminated on day 28, and the explanted tumor was analyzed by flow cytometry, image mass cytometry, and confocal microscopy as described in Materials and Methods.

### Comparative metabolic profiling

The glycolytic states of WT and eEF-2K KO CD8^+^ T cells were analyzed by using an extracellular flux (XF) analyzer (Agilent) using the manufacturer’s protocol with modifications. Briefly, T cells were activated with anti-CD3/CD28 antibodies and cultured for 2 to 4 days in RPMI 1640 medium before assay. Subsequently, 1 × 10^5^ WT or eEF-2K KO T cells were removed from suspension from 48-well plates and transferred to 96-well polylysine-coated Seahorse XF96 Cell Culture Microplate in phenol red–free RPMI 1640–based assay medium. The plate was centrifuged to facilitate the attachment of T cells, and then ECAR was measured following the manufacturer’s protocol.

### Flow cytometric analysis

In vitro cultured CD8^+^ T cells or explanted tumor sections were analyzed by flow cytometry. For intracellular T cell cytokine staining analysis, CD8^+^ T cells were isolated from the explanted tumor and cultured in the RPMI 1640 medium for 4 days before flow cytometric analysis. Mouse CD8^+^ T cells in in vitro culture were stained with fluorochrome-conjugated anti–PD-1, anti-CD27, anti-CD28, and Tim-3 (clone nos. 29F.1A12, RMT3-23, LG 3A10, and 37.51, respectively, BioLegend, San Diego, CA). NF-κB signaling was assessed using antibodies (phospho-IKKα/β, phospho–NF-κB p65, NF-κB p65, and IKKα/β) from an NF-κB pathway sampler kit (catalog no. 9936, Cell Signaling Technology, Danvers, MA). The dead cells were excluded from analysis by using Aqua Zombie NIR staining dye (BioLegend, San Diego, CA) and gated. For in vivo CAR-T cell–based tumor inhibition studies, the explanted tumor was homogenized into single-cell suspension using a GentleMACS mouse tumor dissociation kit (Miltenyi Biotech, Auburn, CA). TILs were then analyzed for their functional profile and infiltration using fluorochrome-conjugated anti-mouse Thy1.2 (CD90.2; BD Biosciences, San Jose, CA), anti–PD-1, anti-CD27, anti-CD28, and anti–Tim-3 antibodies (BioLegend, San Diego, CA). TILs were also sorted using BD FACSAria, and the intracellular cytokine staining of the ex vivo activated CD8^+^ T cells was performed as previously described (*43*). Fluorochrome-conjugated anti–TNF-α (clone no. MP6-XT22), anti–IFN-γ (clone no. XMG1.2), anti–IL-4 (clone no. 11B-11), anti–IL-1α, and anti–IL-2 antibodies (clone no. JES6-5H4) were used for intracellular cytokine staining analysis. All data were acquired using a BD Fortessa X-20 flow cytometer (BD Biosciences, San Jose, CA) with FACSDiva v8 interface.

The data were interpreted and analyzed using FlowJo v10.7. The imaging flow cytometer analysis for in vitro dead-live assay was acquired using an Amnis ImageStream imaging flow cytometer (Luminex Corp, USA). The WT and eEF-2K KO CAR-T cells were stained with CFSE dye and cocultured with MC32 CEA cells. The cells were stained with aqua zombie dye post-assay for determining the live and dead cells, which were represented by pseudo-colored image representation. The graphs were constructed and statistically analyzed in GraphPad Prism 9.

### Tumor imaging and immunohistochemistry

Fresh solid tumor samples were paraffin-embedded and sliced into 4-μm sections with microtome. The prepared slides were processed for H&E staining, fluorescence microscopy, and mass cytometry analysis. The H&E section scoring was done on a scale of 1 to 5, 1 being the least tumor inhibition and 5 being the highest tumor inhibition. The tumor inhibition was calculated by considering the parameters for the number of infiltrating cells into the tumor and total of tumor cells in the xenografts for WT and eEF-2K KO CD8^+^ T cell–treated mice.

### Imaging mass cytometry analysis

Mass cytometry uses heavy metal label–conjugated antibodies, greatly enhancing the deep immunophenotyping analysis of tumor samples. A dimensionality reduction technique, *t*-SNE, was used to analyze several different tumor-associated immune cell markers among the WT CEA-CAR CD8–infused and eEF-2K KO CEA-CAR CD8–infused groups of mice. Heatmap (*11*) plots of the number of cells per neighborhood across the imaged tumor samples were constructed to analyze the local cell densities within individual neighborhoods as described previously (*44*). A subtractive heatmap was constructed from the data to show the differential immune cell relationships among the cell types. Ir191, Er167, Dy162, Er170Sm149, and Yb176 were used for staining DNA, Ki-67 Ag, CD8^+^ T cells, B220 (B cells), CD11b (dendritic cells), and F4/80 (macrophages), respectively.

### Statistical analysis

Multiple Student’s unpaired *t* test or one-way/two-way analysis of variance (ANOVA) was performed to analyze the differences between the groups. For mice survival curve analysis, the Kaplan-Meier method was adopted and compared statistically using the log-rank test in GraphPad Prism. A *P* value of less than 0.05 was considered significant. The illustrations and schematic representations in figures are created by using the BioRender software.

## Notes

View/request a protocol for this paper from *Bio-protocol*.
