# Supplementary material for: Elongation factor-2 kinase is a critical determinant of the fate and antitumor immunity of CD8+ T cells
Source: Sci Adv. 2022 Feb 2;8(5):eabl9783. doi: 10.1126/sciadv.abl9783 (PMC8809536; doi:10.1126/sciadv.abl9783)
Supplement: Supplementary file 1 — Figs. S1 to S7 Table S1 [file sciadv.abl9783_sm.pdf]

Science Advances
AAAS

Supplementary Materials for

Elongation factor-2 kinase is a critical determinant of the fate and antitumor immunity of CD8$^+$ T cells

Jugal Kishore Das, Yijie Ren, Anil Kumar, Hao-Yun Peng, Liqing Wang, Xiaofang Xiong, Robert C. Alaniz, Paul de Figueiredo, Xingcong Ren, Xiaoqi Liu, Alexey G. Ryazonov, Jin-Ming Yang*, Jianxun Song*

*Corresponding author. Email: jyang@uky.edu (J.-M.Y.); jus35@tamu.edu (J.S.)

Published 2 February 2022, *Sci. Adv.* **8**, eabl9783 (2022)
DOI: 10.1126/sciadv.abl9783

This PDF file includes:

Figs. S1 to S7
Table S1

fig. S1

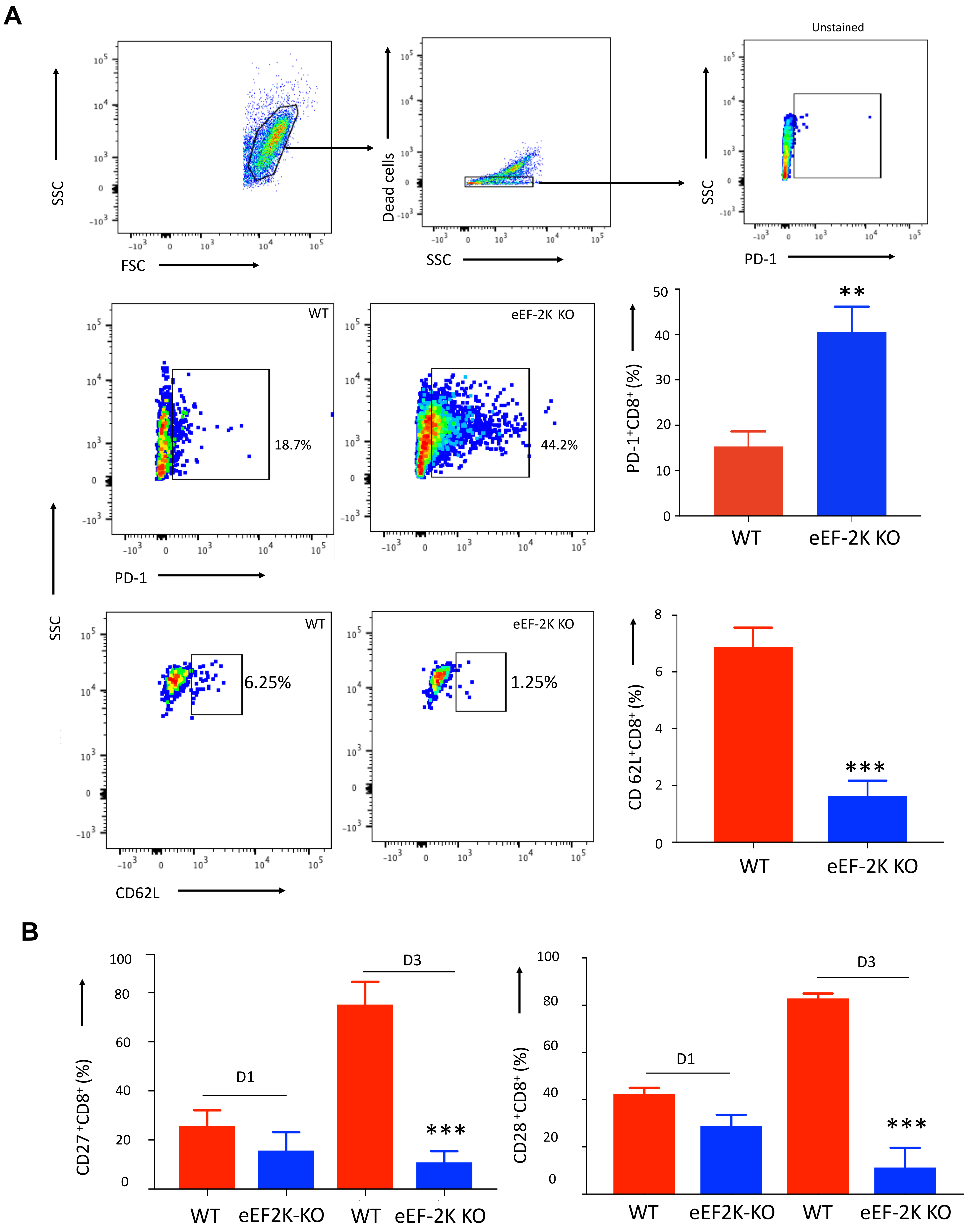

fig. S1. Loss of eEF-2K results in CD8⁺ T cell exhaustion. WT and eEF-2K KO CD8⁺ T cells were activated using anti-CD3/CD28 antibodies for six days. (**A)** Flow cytometric analysis was performed to assess expression of PD-1 and CD62L. The graphical representation of dot-plot analysis in triplicates was shown. (**B**) Graphical representation of flow cytometric assay for CD27 and CD28 on days 1 and 3.

fig. 2S

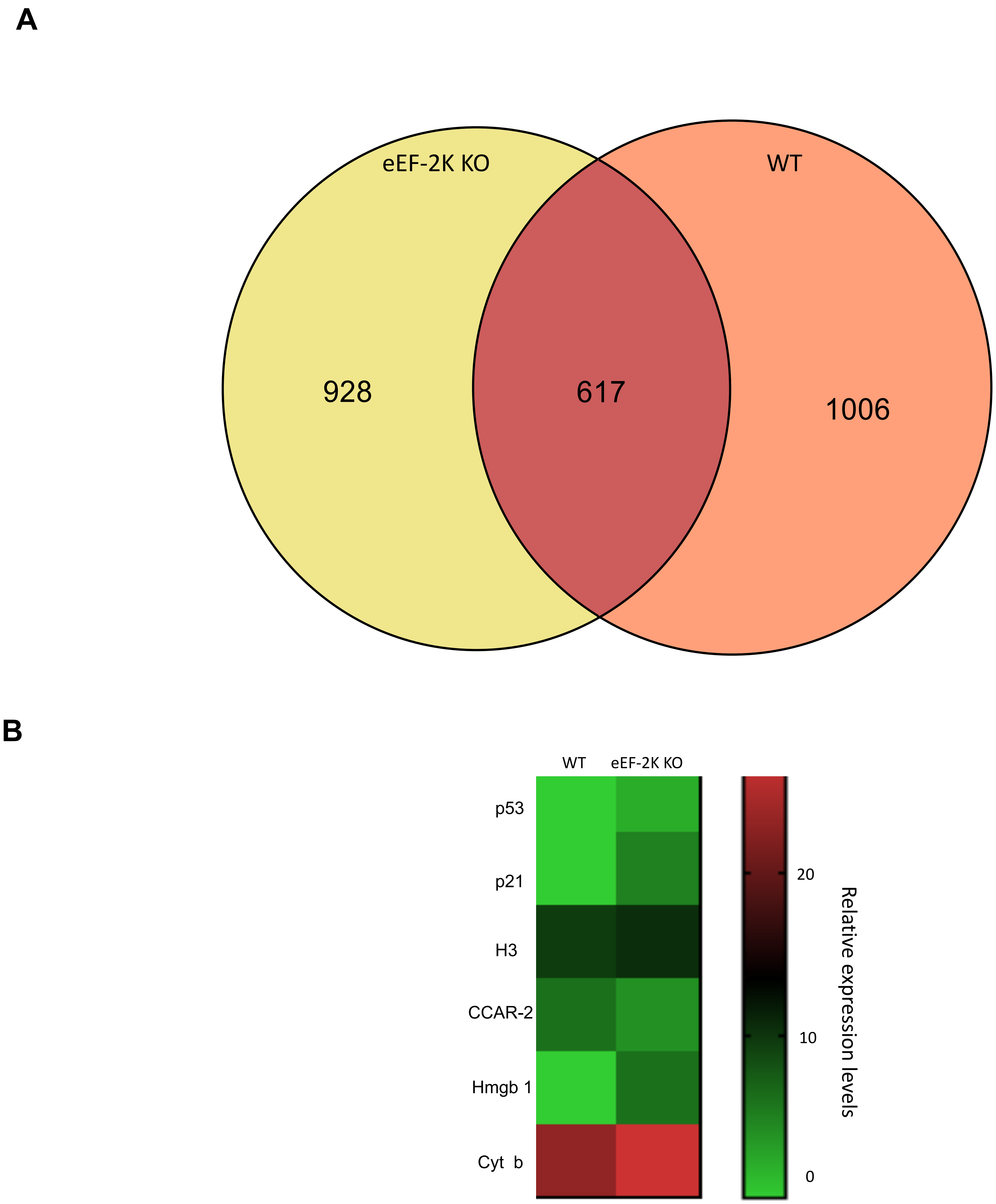

fig. S2. Loss of eEF-2K differentially regulate protein expression. (**A**) LC-MS/MS Proteomics analysis of WT and eEF-2K KO CD8⁺ T cells was represented by Venn-diagram showing differentially expressed proteins. (**B**) Comparative heatmap analysis of senescence and apoptosis markers of WT and eEF-2K KO CD8⁺ T cells derived from the proteomics spectral analysis.

fig. S3

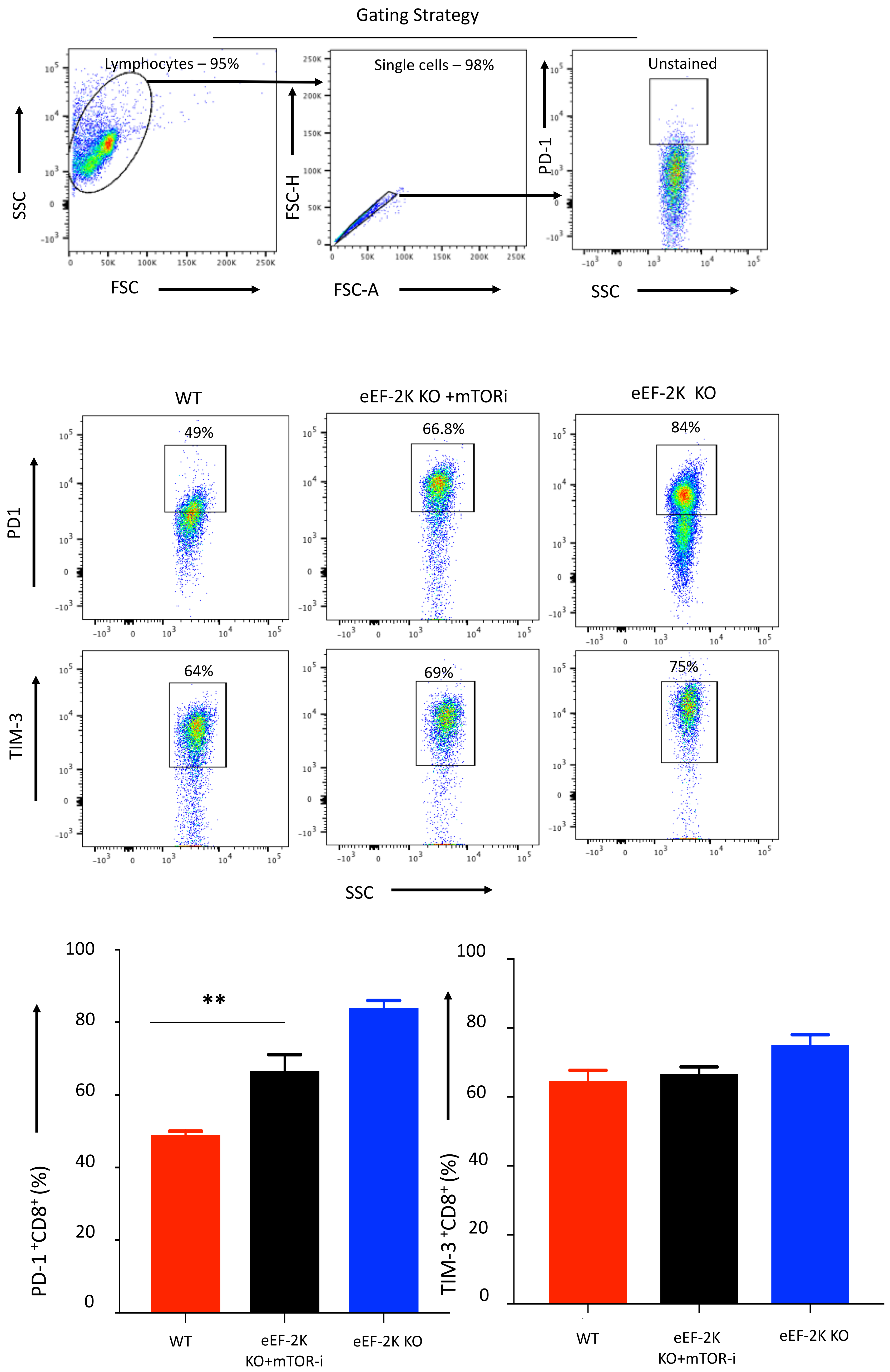

fig. S3. Upregulation of PD-1 and Tim-3 in eEF-2K KO CD8^{+} T cells can be abrogated by rapamycin (mTOR-i). Flow cytometric analysis of PD-1 and Tim-3 expression in WT CD8^{+} T cells, eEF-2K KO CD8^{+} T cells and eEF-2K KO CD8^{+} T cells treated with rapamycin (mTOR-i). Data shown are the graphical representation of the data of 3 independent experiments.

fig. S4

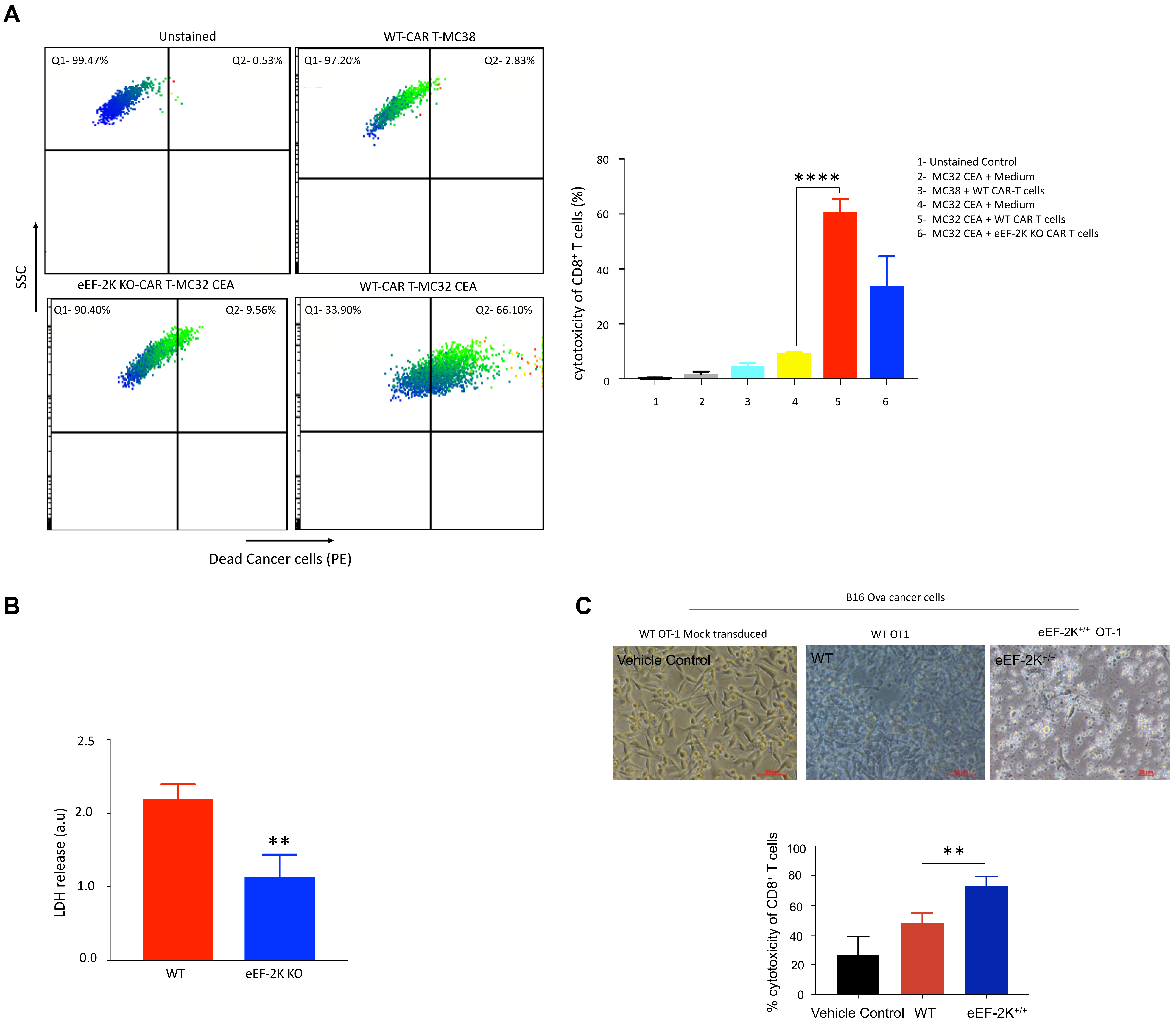

fig. S4. Loss of eEF-2K compromises the cytotoxicity of eEF-2KO CD8⁺ T cells. Control MC38 cells or the MC32 CEA Ag-expressing cells were co-cultured with WT or eEF-2K KO CD8⁺ T cells, and then their cytotoxicity was assessed. (**A**) Representative dot-plots obtained from flow cytometric analysis followed by graphical representation of the dot-plots. (**B**) Graphical representation of LDH release assay of MC32-CEA colon cancer cells co-cultured with WT or eEF-2K KO CD8⁺ T cells. (**C**) The bright field images of the B16-OVA cells co-cultured with control (No OT-I CD8⁺ T cells), WT (non-transduced OT-I CD8⁺ T cells) or eEF-2K-overexpressing OT-I CD8⁺ T cells.

fig. S5

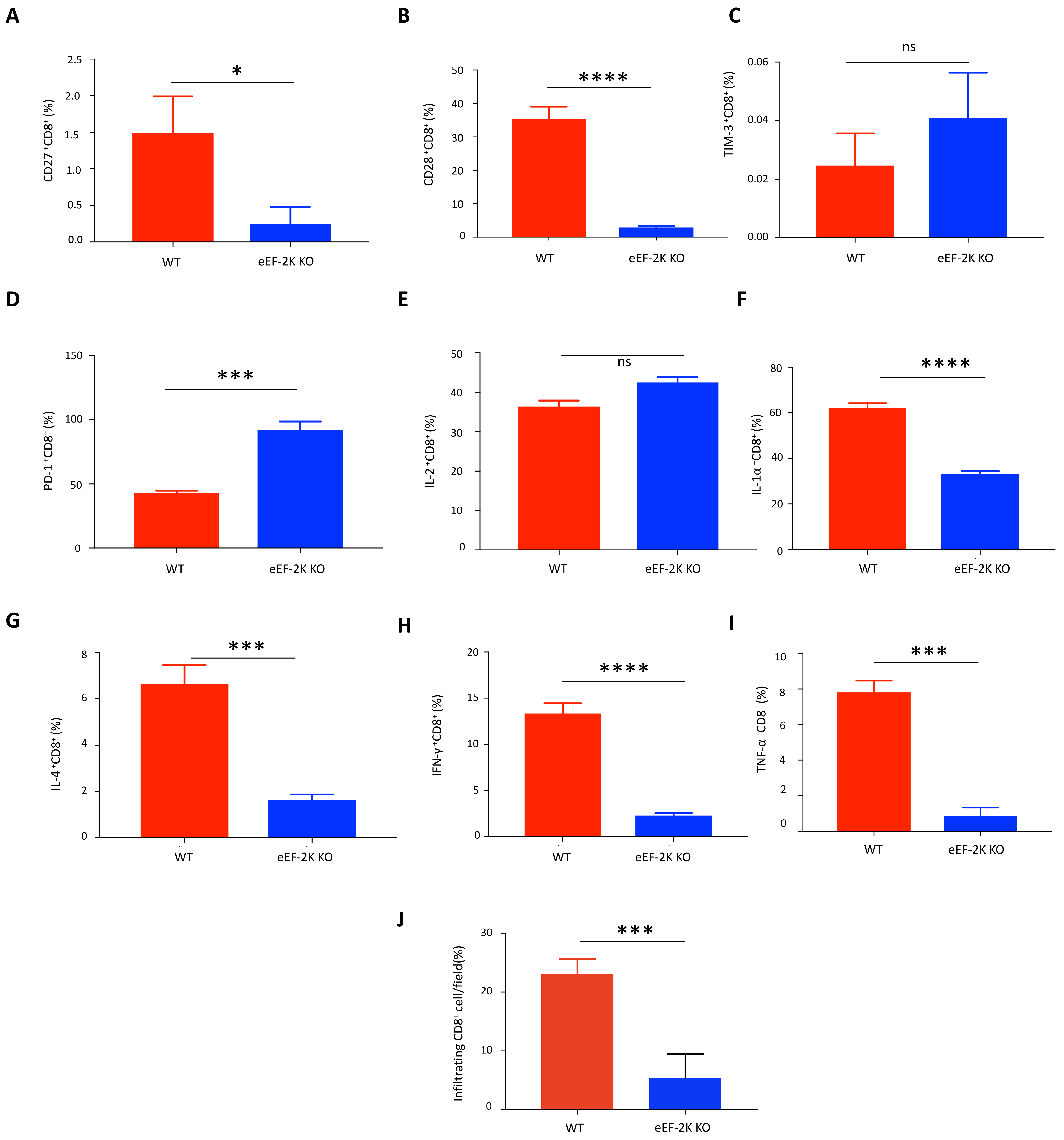

fig. S5. Expression of costimulatory and exhaustion markers by the CEA-specific CAR- CD8⁺ T cells with or without eEF-2K. On day 28 after adoptive transfer, the WT or eEF-2K KO Thy1.2 CD8⁺ T cells (**Figure 6)** were analyzed for the expression of various costimulatory and exhaustion markers, using flow cytometry for graphical representations of expression derived from flow cytometric dot plots. (**A**) CD27. (**B**) CD28. (**C**) TIM3. (**D**) PD-1. The intracellular cytokine expression was also assessed from the WT or eEF-2K KO Thy1.2 CD8⁺ T cells after re-stimulating the cells ex-vivo with anti-CD3/CD28 antibodies. (**E**) IL-2. (**F**) IL-1α. (**G**) IL-4. (**H**) IFN-γ. (**I**) TNF-α. (**J**) Graphical representation of 5 independent fields of tumor infiltrating lymphocytes as observed in confocal microscopy depicted in **Fig. 6G**. Graphical data shown are mean ± SD from values derived from 3 different wells of 3 independent experiments. *p< 0.05; **p< 0.01; ***p< 0.005 ****p< 0.0001.

fig. S6

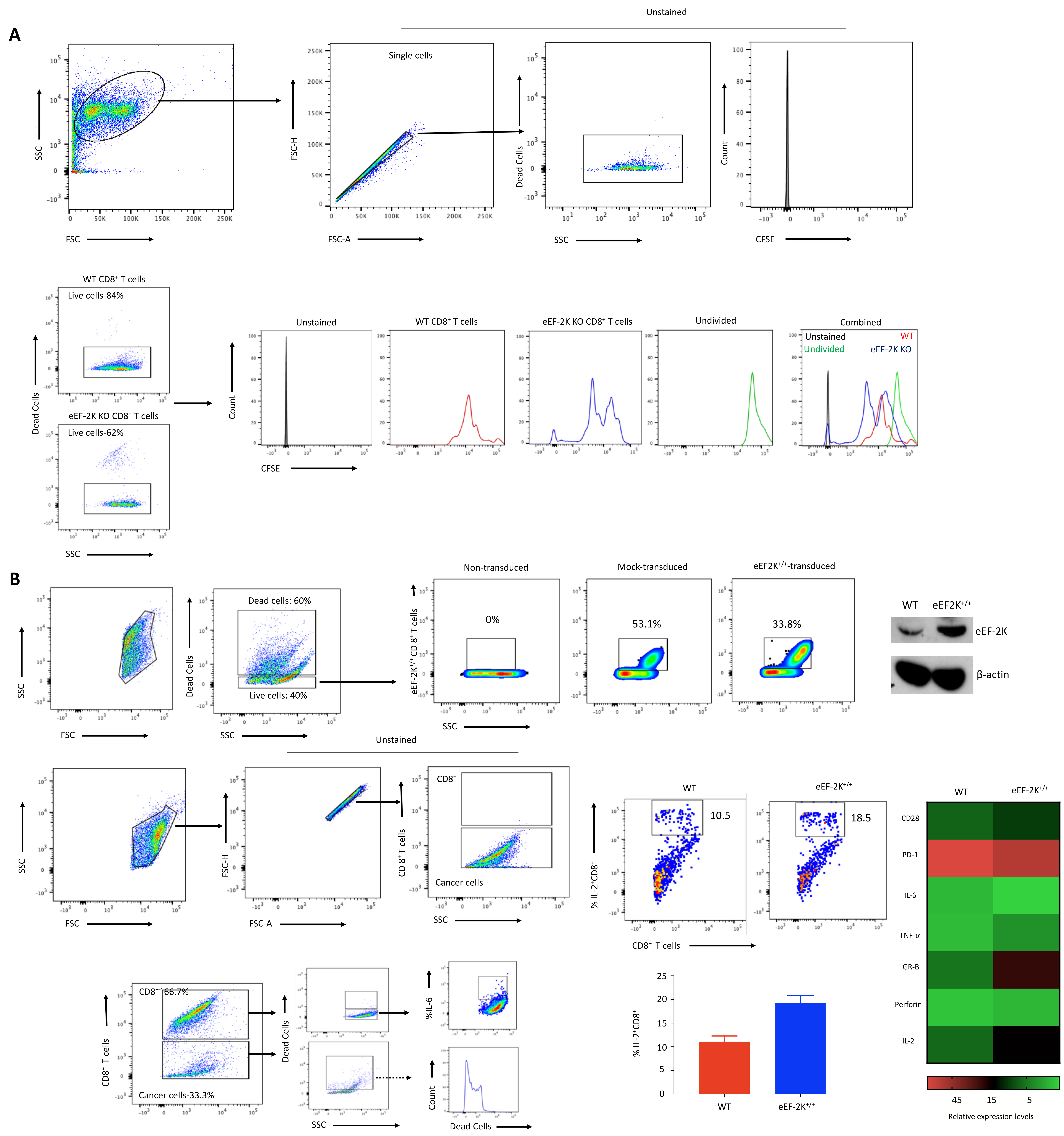

fig. S6. Gating Strategy for proliferation of WT and eEF-2K KO CD8$^+$ T cells. (**A**) The gating strategy for proliferation assay using CFSE staining of WT and eEF-2K KO CD8$^+$ T cells is shown in the figure. The unstained and untransduced controls are shown in the plot. (**B**) The gating strategy for the dead-live staining of B16 cells and the functional analysis of OT-I CD8$^+$ T cells. Western blot analysis showing overexpression of eEF-2K in OT-I CD8$^+$ T cells and heatmap analysis of functional markers expressed on WT and eEF-2K$^{+/+}$ (eEF-2K-overexpressed) OT-I CD8$^+$ T cells co-cultured with B16-OVA cells.

fig. S7

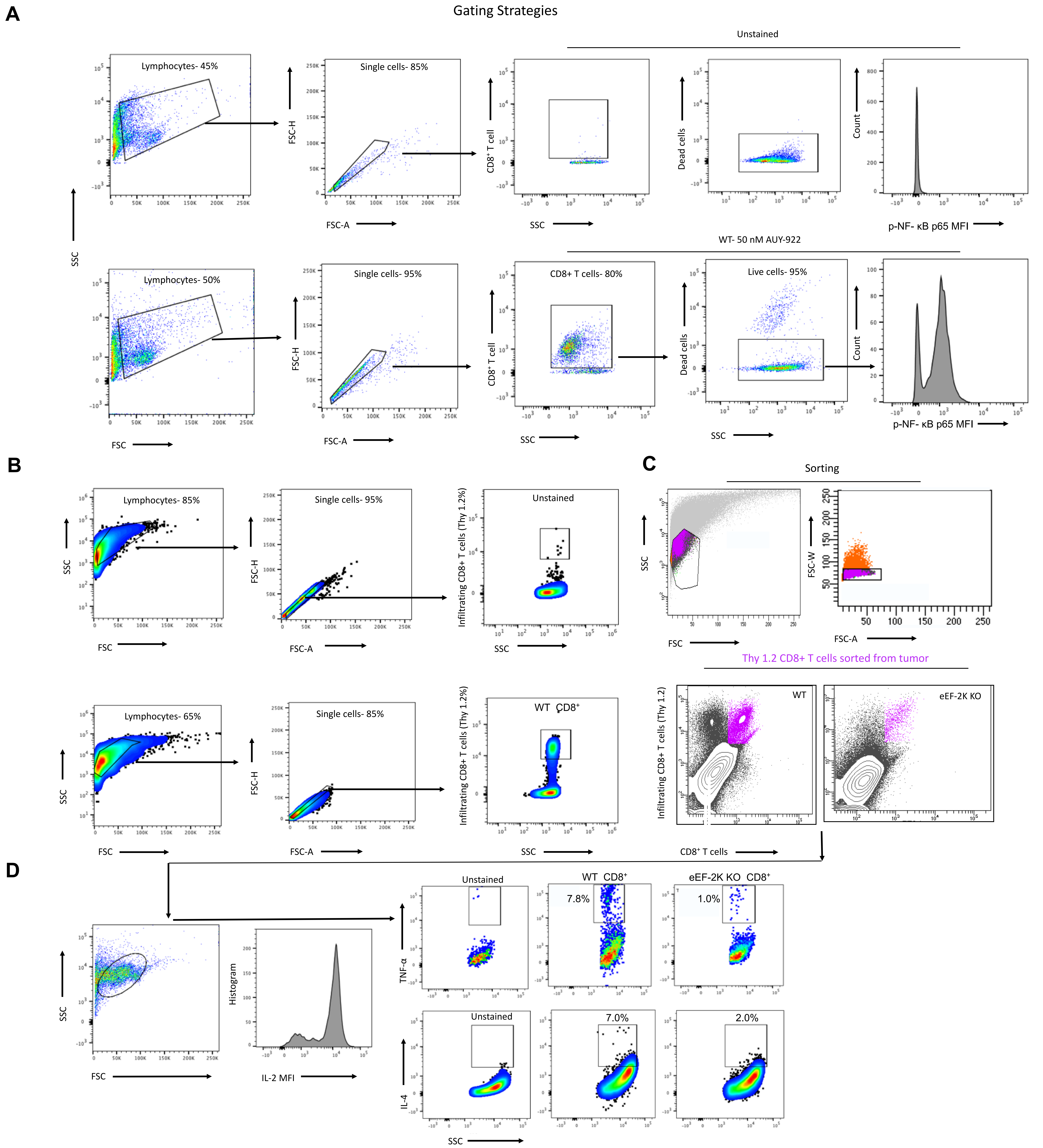

fig. S7. Gating Strategy for flow cytometry assays. The gating strategy for different flow cytometric analysis is shown in the figure. (**A**) Gating strategy for NF-κB activity was the same as described in **Fig 4**. (**B**) Gating strategy for *ex-vivo* tumor explanation experiment was the same as described in **Fig. 6A**. (**C**) Gating strategy for sorting of tumor infiltrating Thy1.2 CD8$^+$ T cells followed by representative dot-plot analysis of infiltrating CD8$^+$ T cells. (**D**) IL-2 cytokine analysis (MFI) and representative dot plots for TNF-α and IL-4 cytokine staining are shown in the figure.

Table S1. Antibodies used in Western blot and Flow cytometric Analysis

Antibodies (Western blot)	Cat no.	Secondary	Company
Akt	680302	Anti-mouse HRP	BioLegend
p-Akt	550747	Anti-mouse HRP	BD Bioscience
mTOR	A301-143 A	Anti-rabbit HRP	Bethyl lab
p-mTOR	2971	Anti-rabbit HRP	Cell Signaling
p-RPS6Kb	608602	Anti-mouse HRP	BioLegend
eEF-2K	#3692	Anti-mouse HRP	Cell signaling
Antibodies (Flowcytometry)	Cat no.	Fluorophore	Company
PD-1	135210	APC	BioLegend
Tim-3	119727	BV-711	BioLegend
CD27	124210	PE	BioLegend
CD28	102127	BV 421	BioLegend
IL-2	503810	APC	BioLegend
IL-1-α	14-7011-81	FITC	e-Bioscience
IFN- γ	505805	FITC	BioLegend
TNF-α	506327	BV-421	BioLegend
IL-4	504103	PE	Biolegend
CD8	300906	FITC	BioLegend
CD90.2 (Thy1.2)	561974	APC	BD Biosciences
NF-κB sampler kit	9936	Various	Cell signaling
